# Consolidation therapy with autologous stem cell transplantation after remission of induction chemotherapy prolongs the survival of patients with peripheral T-cell lymphoma

**DOI:** 10.3389/fimmu.2024.1382189

**Published:** 2024-05-10

**Authors:** Wulipan Fulati, Jiexian Ma, Min Wu, Wensi Qian, Pingping Chen, Yingwei Hu, Mingyue Chen, Yu Xu, Zilan Huang, Hongdi Zhang, Yanhui Xie, Lin Shen

**Affiliations:** Department of Hematology, Huadong Hospital Affiliated with Fudan University, Shanghai, China

**Keywords:** peripheral T cell lymphoma, autologous hematopoietic stem cell transplantation, pegylated liposomal doxorubicin, survival analysis, prognosis

## Abstract

**Background:**

There was little evidence of autologous stem cell transplantation (ASCT) as consolidation therapy after remission of induction for patients with Peripheral T-cell lymphoma (PTCL). In this study, we conducted a comparative analysis of real-world survival outcomes between consolidation therapy and observation in patients with PTCL.

**Methods:**

A total of 92 patients with peripheral T-cell lymphoma (PTCL) who were admitted to the Department of Hematology, Huadong Hospital Affiliated with Fudan University from January 2013 to April 2019 were divided into two groups based on whether they were treated with high-dose therapy (HDT) followed by autologous hematopoietic stem cell transplantation (ASCT): ASCT as consolidation therapy (n=30) and observation (n=62). Clinical characteristics, treatment patterns, and survival outcomes were analyzed between the two groups. Univariate and Cox multivariate regression analyses were also performed to detect prognostic factors of survival.

**Results:**

With a median follow-up time of 41 months, the median overall survival (OS) of peripheral T-cell lymphoma patients treated with ASCT was not reached; the median progression-free survival (PFS) was 77.0 months, which was much higher than that of patients without ASCT (*p*<0.003 for OS, p=0.015 for PFS). Subgroup analysis found that patients with high risks benefited more from ASCT. Combination with hemophagocytic lymphohistiocytosis (HLH) (*p*<0.001), clinical stage more than III (*p*=0.014), IPI score above 3 (*p*=0.049), and bone marrow involvement (*p*=0.010) were the independent prognostic factors significantly associated with worse OS and PFS. Additionally, pegylated liposomal doxorubicin (PLD)–containing chemotherapy regimen could bring a higher overall response rate (ORR) and prolong the survival of patients with PTCL who underwent ASCT.

**Conclusion:**

ASCT may improve the long-term survival of patients with PTCL as consolidation therapy after achieving complete or partial remission of induction treatment, particularly for those with high risks. The chemotherapy regimen containing pegylated liposomal doxorubicin may induce deeper remission than traditional doxorubicin in PTCL. It is crucial to identify the specific groups most likely to benefit from upfront ASCT.

## 1 Introduction

Lymphomas are malignant tumors originating from the lymphopoietic system. Compared with B-cell lymphoma, Peripheral T-cell lymphoma (PTCL) is more aggressive, and the treatment efficacy for PTCL is suboptimal, leading to a poor prognosis (1). PTCL is more prevalent on the Asian continent and accounts for approximately 10%–20% of cases of NHL, warranting further concern and research (2). The first-line treatment for PTCL often involves induction chemotherapy containing anthracyclines followed by autologous stem cell transplantation (ASCT) in many countries, although not confirmed by large prospective randomized controlled studies. Prospective data from studies of upfront ASCT consolidation in PTCL (excluding ALK-positive ALCL) have shown that this approach can prolong patient survival (3, 4). However, this recommendation was based on several single-arm prospective or retrospective studies, and the conclusions drawn from them were contradictory (5–9). Therefore, the role of ASCT as consolidation therapy for PTCL needs further clarification and validation. Additionally, identifying predictors of efficacy to screen for populations that would benefit more from ASCT consolidation is worth exploring.

Herein, we conducted this retrospective study to examine the effectiveness and safety of ASCT for the treatment of T-cell lymphoma and to define prognostic factors predicting survival benefit using real-world data.

## 2 Materials and methods

### 2.1 Study design

This was a single-center, retrospective study conducted at the Department of Hematology, Huadong Hospital affiliated with Fudan University (Shanghai, China). Clinical records of patients diagnosed with peripheral T-cell lymphoma from January 2013 to April 2019 were collected and analyzed. The study was approved by the Huadong Hospital ethics committee (2024K150). Clinical variables included gender, age, clinical presentation, pathological type, clinical stage, Eastern Cooperative Oncology Group (ECOG) score, International Prognostic Index (IPI), Epstein-Barr virus(EBV) infection status, hemophagocytic lymphohistiocytosis (HLH), treatment regimen, response, adverse effects, progression-free survival (PFS), and overall survival (OS).

### 2.2 Inclusion and exclusion criteria

All patients were diagnosed following the 2008 WHO classification criteria for lymphoma hematopoietic tissue tumors (10). Inclusion criteria were as follows: 1) clear diagnosis of PTCL by histopathology, including peripheral T-cell lymphoma, not otherwise specified (PTCL, NOS), anaplastic large-cell lymphoma [ALCL, with or without expression of the anaplastic lymphoma kinase (ALK+, ALK-)], angioimmunoblastic T-cell lymphoma (AITL), hepatosplenic T-cell lymphoma; 2) received standard treatment at our hospital and achieved complete response (CR) or partial response (PR) to induction therapy. All patients who underwent upfront transplantation were required to be younger than 65 years old and must have an Eastern Cooperative Oncology Group performance status score of <2; 3) complete clinical data, including staging assessment tests at the time of consultation, evaluation of efficacy during treatment, and regular follow-up examinations. Exclusion criteria were as follows: patients with other malignant tumors, those with active infections, or patients with vital organ dysfunctions such as abnormal liver, kidney, and heart functions. Patients were also excluded if they had severe concomitant medical or psychiatric illnesses or if they were seropositive for human immunodeficiency virus.

### 2.3 Treatment regimen

Patients were treated based on the NCCN guidelines by pathological staging, stage, and general condition. The dose of chemotherapy drugs or dosing cycles could be adjusted depending on treatment toxicities and patient tolerance. The first-line treatment regimen for T-cell lymphoma in our hospital is CHOP (doxorubicin 50 mg/m2 d1, cyclophosphamide 750 mg/m2 d1, vincristine 1.4 mg/m2 d1, prednisone 100mg d1-5) regimen and CDOP (cyclophosphamide 750 mg/m2 d1, pegylated liposomal doxorubicin (PLD, Shanghai Fudan-Zhangjiagang Bio-Pharmaceutical Co., Ltd, China), 20-40 mg/m2 d1, vincristine 1.4 mg/m2 d1, prednisone 100mg d1-5). We chose pegylated liposomal doxorubicin (PLD) or traditional doxorubicin according to the patient’s willingness, economic situation, and presence of chronic disease, especially heart function failure.

After standardized chemotherapy treatment with at least 4–6 cycles and remission (CR+PR) evaluated by 18-fluorodeoxyglucose positron emission tomography/computed tomography (PET/CT), patients were divided into two groups based on whether they underwent ASCT. The choice of ASCT depended on the patient’s age, physical status, disease status, and willingness: (1) the non-ASCT group (62 patients); (2) the ASCT group (30 patients).

All patients in this study in our center received HSCT with a conditioning regimen: Chemo-mobilization (cyclophosphamide(CTX) 30mg/kg + Etoposide(VP-16) 15mg/kg) according to institutional standards of care. G-CSF mobilization was given to patients during the beginning of myelosuppression recovery, with 5ug/kg G-CSF administered daily for 6 days. BEAM (carmustine, 300 mg/m2, -7d; etoposide, 200 mg/m2/d, -6d~-3d; cytarabine, 200 mg/m2/d, -6d~-3d; melphalan 140 mg/m2, -2d) was used as high-dose therapy (HDT) for patients. And in our center, the graft source is PBSC. Collection of blood grafts: Apheresis was initiated from the fifth day and continued daily for 2 days until the predetermined minimal target yield (mononuclear cells ≥2.0 × 10^8/kg and CD34+ cells ≥1.0 × 10^6/kg) was achieved. The apheresis was carried out initially with the Spectra AutoPBSC (Terumo BCT, Lakewood, Colorado) apheresis machine. The number of CD34+ cells in each apheresis bag was measured by flow cytometry using an International Society of Hemotherapy and Graft Engineering protocol with a single platform method at the stem cell laboratory of each apheresis center. The antibodies against the following cell surface markers were used: CD34, CD38, CD133, and CD45. All antibodies were provided by Beckman Coulter. The graft was infused on Day 0 without freezing. Filgrastim was administered after the graft infusion.

### 2.4 Evaluation of efficacy and toxic side effects

#### 2.4.1 Response criteria

Responses were categorized as complete remission (CR), partial remission (PR), stable disease (SD), disease progression (PD), and overall response rate (ORR=CR+PR) after completion of six cycles of treatment using the revised IWG criteria based on CT or PET/CT.

#### 2.4.2 Endpoint and follow-up

All patients were followed up on an outpatient basis or by telephone until December 31, 2021, and survival was calculated from the date of pathological confirmation. The duration of response (DOR) was defined as the time from the first evaluation of response as CR or PR to the first evaluation of PD or death from any cause. Overall survival (OS) time was defined as the time interval from the date of diagnosis to the date of death from any cause or the date of the last follow-up visit. Progression-free survival (PFS) time was defined as the time interval from the date of patient diagnosis to the date of disease progression, death, or final follow-up. In this study, OS and PFS were calculated monthly.

#### 2.4.3 Safety

All adverse events (AE) and serious adverse events (SAE) were collected and graded according to the Common Terminology Criteria for Adverse Events (CTCAE-Version 4.0).

### 2.5 Statistical analysis

Descriptive statistics were used to present patient and treatment characteristics. Categorical variables were presented as n (%) and continuous variables were presented as mean ± standard deviations or median (interquartile range, IQR). A comparison of the characteristics of the two groups was performed using Fisher’s exact test or Pearson’s chi-squared test for categorical variables and Student’s t-test for continuous variables. Kaplan-Meier survival analysis was used to evaluate the OS and PFS, with the log-rank test used to compare the survival rates between the two groups. Hazard ratios (HRs) and corresponding 95% confidence intervals (CIs) were derived from Cox proportional-hazard regression models. To investigate the prognostic factors of PFS and OS, univariate and multivariate Cox analyses were conducted. A two-tailed p-value < 0.05 was considered statistically significant. SPSS 19.0 software was utilized for data comparison between the two groups, and graphs were drawn using Prism Version 8.0.1 (San Diego, CA, USA).

## 3 Results

### 3.1 Patient and treatment characteristics

Out of the 92 patients included in this analysis, 30 were in the ASCT group and 62 were in the non-ASCT group. The median age for all patients was 49.5 years (range: 18–83) for the entire cohort. The clinical characteristics of patients in the two groups are summarized in **Table 1**. The median age of patients in the ASCT group was younger than that in the non-ASCT group (36 vs. 52 years; *p*<0.001), and a higher proportion of patients with HLH(*p*<0.001) was observed in the ASCT group compared to the non-ASCT group. However, there were no significant differences in gender, IPI score, EBV infection rate, the presence of elevated lactate dehydrogenase (LDH), B2 microglobulin, or bone marrow involvement between the two groups.

**Table 1:** Patient characteristics at baseline and treatment characteristics.

Characteristics	ASCT group	Non-ASCT group	*P*
**Mean-age, median years (range)**	36 (19-62)	52 (15-83)	<0.001
**Male sex, n (%)**	18 (60.0)	45 (72.6)	0.051
**IPI score at diagnosis, n (%)**			0.370
<3	11 (36.7)	19 (30.6)	
>=3	19 (63.3)	43 (69.4)	
**Ann Arbor stage, n (%)**			0.329
I/II	4 (13.3)	11 (17.7)	
III/IV	26 (86.7)	51 (82.4)	
**EBV infection, n (%)**			0.119
Yes	14 (6.7)	36 (58.1)	
No	16 (53.3)	26 (41.9)	
**LDH, n (%)**			0.253
High	16 (53.3)	38 (61.3)	
Normal	14 (46.7)	24 (38.7)	
**Beta2-microglobulin, n (%)**			0.663
High	18 (60.0)	39 (62.9)	
Normal	12 (40.0)	23 (37.1)	
**BM involvement, n (%)**			0.152
Yes	20 (66.7)	32 (51.6)	
No	10 (33.3)	30 (48.4)	
**HLH, n (%)**			<0.001
Yes	19 (63.3)	27 (43.5)	
No	11 (36.7)	35 (56.5)	
**Pathological type, n (%)**			0.461
PTCL-NOS	17 (56.7)	36 (58.1)	
ALCL-ALK+	2 (6.7)	2 (3.2)	
ALCL-ALK-	3 (10.0)	5 (8.1)	
AITL	6 (20.0)	13 (21.0)	
Hepatosplenic	2 (6.6)	6 (9.7)	
**Induction treatment regimen, n (%)**			0.777
CHOP	13 (43.3)	30 (48.4)	
CDOP	17 (56.7)	32 (51.6)	

IPI, International Prognostic Index; EBV, Epstein-Barr virus; ECOG, Eastern Cooperative Oncology Group; LDH, lactate dehydrogenase; BM, bone marrow; HLH, hemophagocytic lymphohistiocytosis; PTCL-NOS, peripheral T-cell lymphoma, not otherwise specified; ALCL, anaplastic large-cell lymphoma; ALK, anaplastic lymphoma kinase; AITL, angioimmunoblastic lymphoma; CHOP, cyclophosphamide, doxorubicin, vincristine, and prednisone; CDOP, cyclophosphamide, Pegylated liposomal doxorubicin, vincristine, and prednisone.

Regarding the induction treatment regimen, 13 patients (43.4%) in the ASCT group and 30 patients (48.4%) in the non-ASCT group received the CHOP regimen, and no significant difference was observed (*p*=0.777). All patients achieved complete remission (CR) or partial remission (PR) after receiving chemotherapy.

### 3.2 Survival analysis

**Figure 1:**
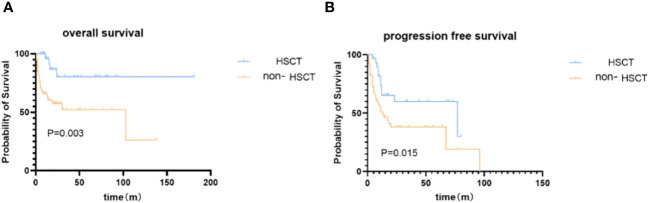
Kaplan–Meier survival analysis of patients in ASCT and non-ASCT groups. **(A)** overall survival. **(B)** progression-free survival.

With a median follow-up time of 41 months (range: 1–217), the median overall survival (OS) was 103.0 months (95% confidence interval [95%CI]: 0.0-223.2), with a 5-year OS rate of 62.1%. The median progression-free survival (PFS) was 18.0 months (95%CI: 0.00-50.749), with a 3-year PFS rate of 54.3% (as shown in **Figure 1**) in the entire cohort.

We then investigated the impact of ASCT on survival. At the end of the follow-up time, the disease relapse rate following transplant is 33.3%; NRM after ASCT is 1 case. A significantly higher OS rate at 5 years was observed in the ASCT group [80.5% (95%CI: 80.3%-80.7%) vs 52.2% (95%CI: 52.0%-52.4%), **Figure 1**], with a median OS not reached (95%CI: not estimated) in the ASCT group compared to 87.0 months (95%CI: 3.6-202.4) in the non-ASCT group (*p*=0.003). The PFS was also significantly better in the ASCT group [59.8% (95%CI: 59.6%-60.0%) vs 38.2% (95%CI: 38.1%-38.3%)] at 3 years, with a median PFS of 77.0 months (95%CI: 0.1-153.9) in the ASCT group compared to 13.0 months (95%CI: 5.1-20.9) in the non-ASCT group (*p*=0.015).

### 3.3 Prognosis factors

In the univariate analysis (**Table 2**), IPI score (*p*=0.014), LDH elevation (*p*=0.024), B2-MGs elevation (*p*=0.009), bone marrow involvement (*p*=0.002), combination with HLH (*p*<0.001), and upfront auto-HSCT (*p*=0.007) were significantly associated with OS (*p*<0.05). Additionally, B2-MGs elevation (*p*=0.047), bone marrow involvement (*p*=0.002), combination with HLH (*p*=0.001), and ASCT consolidation therapy (*p*=0.020) were significantly associated with PFS (*p*<0.05).

**Table 2:** Comparison of the effect of treatment in patients with different clinical features.

	OS				PFS			
	Univariable		Multivariable		Univariable		Multivariable	
	HR (95%CI)	*P*	HR (95%CI)	*P*	HR (95%CI)	*P*	HR (95%CI)	*P*
**Age, >=60**	1.11 (0.50-2.46)	0.803	0.64 (0.25-1.64)	0.352	0.94 (0.50-1.80)	0.854	0.58 (0.27-1.26)	0.169
**IPI score, >=3**	0.34 (0.15-0.81)	**0.014**	0.99 (0.36-2.70)	0.976	0.79 (0.44-1.43)	0.432	2.17 (1.00-4.69)	**0.049**
**Ann arbor stage,** III-IV	6.10 (0.83-44.89)	0.076	5.50 (0.59-51.55)	0.136	2.57 (0.92-7.18)	0.073	4.80 (1.38-16.67)	**0.014**
**LDH,** high	2.68 (1.14-6.30)	**0.024**	1.03 (0.30-3.53)	0.963	1.49 (0.81-2.73)	0.198	0.70 (0.29-1.77)	0.441
**B2-MG,** high	3.13 (1.33-7.35)	**0.009**	0.84 (0.25-2.77)	0.769	1.86 (1.01-3.41)	**0.047**	0.94 (0.39-2.25)	0.887
**BM involvement**	3.50 (1.59-7.74)	**0.002**	2.16 (0.79-5.94)	0.135	2.64 (1.45-4.83)	**0.002**	2.82 (1.28-6.20)	**0.010**
**EBV infection**	1.78 (0.83-3.85)	0.141	0.69 (0.29-1.63)	0.399	1.61 (0.88-2.93)	0.120	0.82 (0.42-1.60)	0.558
**with HLH at diagnose**	5.38 (2.50-11.61)	**<0.001**	6.00 (2.27-15.84)	**<0.001**	3.04 (1.62-5.72)	**0.001**	3.81 (1.74-8.34)	**<0.001**
**ASCT**	0.24 (0.02-0.68)	**0.007**	0.18 (0.05-0.60)	**0.005**	0.44 (0.22-0.88)	**0.020**	0.21 (0.09-0.50)	**<0.001**

The bold value was p < 0.05 which was considered statistically significant.

In the multivariate analysis, a combination with HLH (*p*<0.001) and ASCT consolidation therapy (*p*=0.005) were significantly associated with OS (*p*<0.05). Furthermore, clinical stage (*p*=0.014), IPI score (*p*=0.049), bone marrow involvement (*p*=0.010), combination with HLH (*p*<0.001), and ASCT consolidation therapy (*p*<0.001) were significantly associated with PFS (*p*<0.05).

Additionally, we conducted univariate analysis to evaluate the impact of ASCT on OS by subgroups (**Table 3**). A single-factor stratification study revealed that compared with the non-ASCT group, ASCT as a consolidation regimen was beneficial in increasing the long-term OS rate, particularly for males [*p* = 0.001, HR 0.15 (95% CI: 0.04–0.66)], patients with stage III or IV disease [*p* = 0.002, HR 0.15 (95% CI: 0.05-0.50)], patients with an IPI score ≥ 3 [*p* = 0.050, HR 0.13 (95% CI: 0.02-1.00)], those with high LDH [*p*= 0.024, HR 0.24 (95% CI: 0.07-0.83)] and high B2-MG [*p*= 0.034, HR 0.21 (95% CI: 0.05-0.89)], patients with bone marrow involvement [*p*= 0.033, HR 0.20 (95% CI: 0.05-0.88)], and EBV infection [*p*= 0.033, HR 0.20 (95% CI: 0.05-0.88)]. ASCT demonstrated a tendency to prolong OS in patients with HLH at diagnosis [*p*=0.052, HR 0.23 (95% CI: 0.05-1.02)]. Details are presented in **Table 3**.

**Table 3:** Forest plot of overall survival stratified by baseline characteristics.

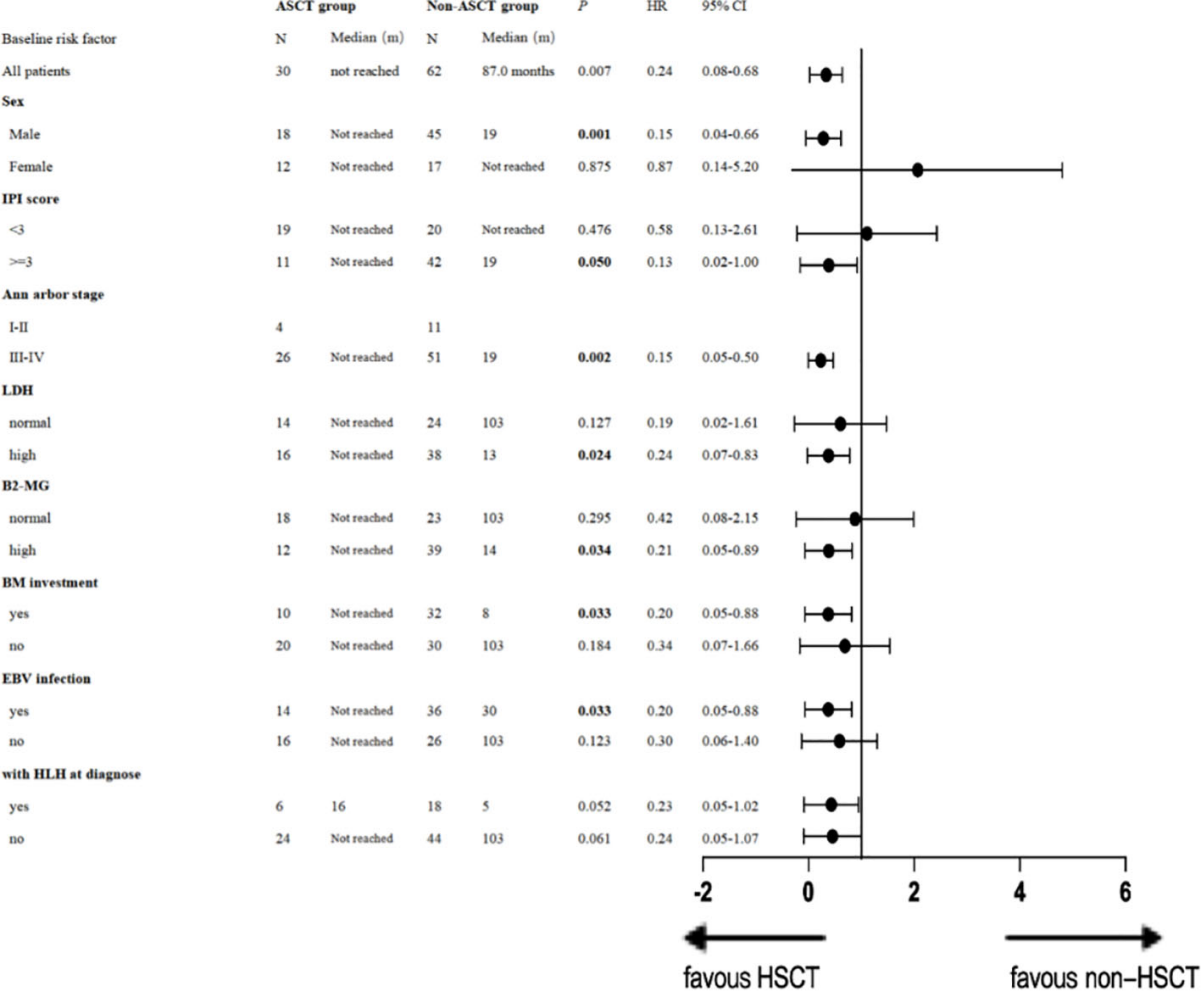

For PFS, univariate analysis by subgroups revealed that patients with stage III or IV disease [*p*= 0.005, HR 0.35 (95% CI: 0.17-0.72)], and those with an IPI score ≥ 3 [*p*= 0.028, HR 0.20 (95% CI: 0.05-0.84)] and high LDH [*p*=0.027, HR 0.33 (95% CI: 0.13-0.88)] would benefit from ASCT. Details are presented in **Table 4**.

**Table 4:** Forest plot of progression-free survival stratified by baseline characteristics.

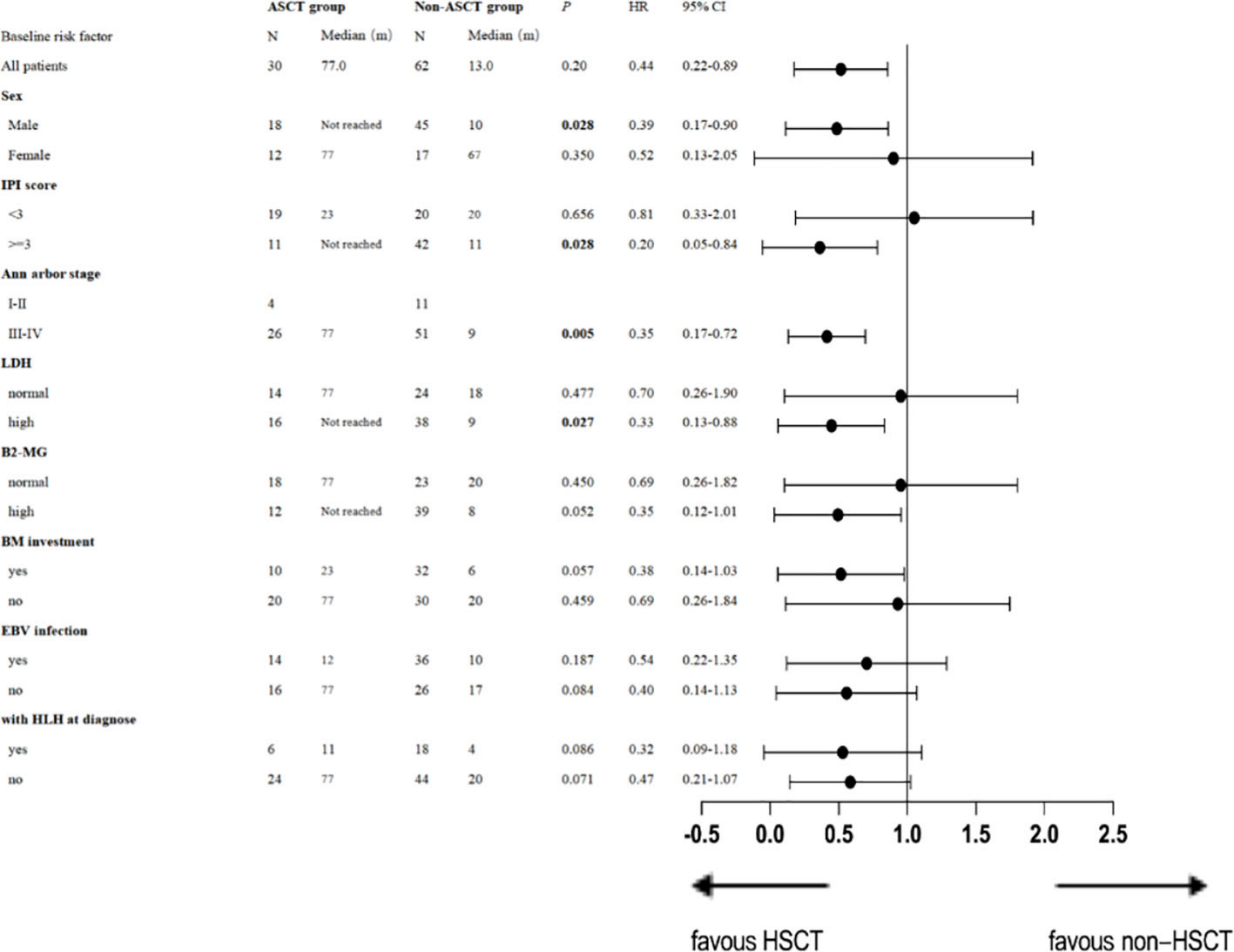

### 3.4 Additional analysis of patients who received ASCT

**Figure 2:**
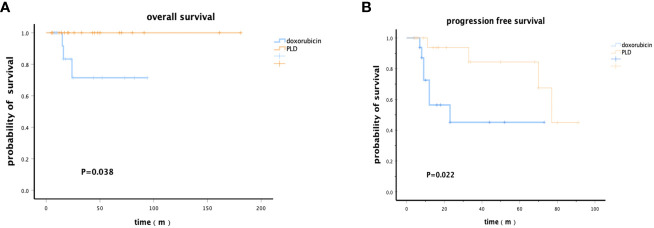
Kaplan–Meier survival analysis of patients who received PLD or doxorubicin chemotherapy regimens followed by ASCT. **(A)** Overall survival. **(B)** Progression-free survival.

Further analysis was conducted in patients who received ASCT to determine which type of patients could benefit more. Patients treated with the PLD-containing chemotherapy as an induction regimen had significantly higher OS rates at 5 years [91.7% (95%CI: 91.5%-91.9%) vs. 71.4% (95%CI: 71.1%-71.7%)] and PFS rates at 3 years [76.0% (95%CI: 75.8%-76.2%) vs. 45.1% (95%CI: 44.8%-45.4%)] than those treated with traditional doxorubicin (*p*OS=0.038, *p*PFS=0.022) (**Figure 2**).

To further investigate the reasons for these results, we analyzed the overall response rate (ORR) of induction therapy in patients before ASCT. Patients in the PLD–containing chemotherapy regimen group had a higher ORR [82.6% (95%CI: 59.8%-102.7%) vs 60.9% (95%CI: 27.5%-86.8%), p=0.021]. Among them, 7 patients achieved complete remission (CR) in the PLD–containing chemotherapy regimen group, with a CR rate of 50.0%(95%CI: 20.0%-80.0%). In the CHOP group, 3 patients achieved CR with a CR rate of 37.5%(95%CI: -5.80%-80.8%). Although the CR rate of patients in the CDOP group was higher, the difference was not statistically significant (p=0.077), indicating a tendency towards benefit among patients with PTCL in terms of chemotherapy efficacy, resulting in better survival outcomes.

### 3.5 Safety

Safety during therapy was assessed for the 30 patients who underwent HSCT. All adverse events are presented in **Table 5**. The most common adverse events with grade 3-4 severity were febrile neutropenia (100%) and nausea and vomiting (76.7%). There were no occurrences of veno-occlusive disease/sinusoidal obstruction syndrome (VOD/SOS, 0%). Additionally, no cardiac adverse events were reported in the ASCT group during the long-term follow-up periods, as monitored by myocardial markers and cardiac echography.

**Table 5:** Adverse Events related to the regimen (n=30).

Adverse events	Grade 1-2, N (%)	Grade 3-4, N (%)
Mucositis (Grade III or IV)	3 (10.0%)	2 (6.7%)
Febrile neutropenia (Grade IV)	0 (0)	30 (100%)
Nausea/vomiting	30 (100%)	23 (76.7%)
Cardiac toxicity(tachycardia, cardiac arrest, heart failure)	10 (33.3%)	2 (6.7%)
VOD/SOS	0	0 (0)
CNS reactions	2 (6.7%)	0 (0)

CNS, central nervous system; VOD/SOS, veno-occlusive disease/sinusoidal obstruction syndrome.

## 4 Discussion

In this study, we collected real-world clinical data from 92 patients at Huadong Hospital affiliated with Fudan University. With similar baseline clinical characteristics between the ASCT group and non-ASCT group, multivariate analysis demonstrated a significant benefit of consolidation therapy with ASCT after remission of induction in PTCL, (median PFS of 77.0 months and a median OS not reached).

Peripheral T-cell lymphoma (PTCL), a heterogeneous disease with multiple pathological subtypes, is characterized by its aggressiveness and propensity for drug resistance and disease progression, resulting in unfavorable survival outcomes (11). Despite CHOP and CHOP-like chemotherapy regimens containing anthracyclines being the standard first-line treatment for TCL, their efficacy remains unsatisfactory (12). Autologous stem cell transplantation (ASCT), shown to significantly improve patient survival compared to chemotherapy alone in multiple studies (13–15), has demonstrated 5-year overall survival (OS) and progression-free survival (PFS) rates of 80% and 67%, respectively, in the consolidation set after the first remission in a retrospective study (16). Similar results have been observed in several prospective single-arm trials (4–7). However, conflicting results have emerged from another study (9), and the absence of randomized clinical trials has precluded the establishment of a definitive consensus regarding the role of ASCT as upfront consolidation therapy for PTCL patients.

In the present study, we directly compared the survival outcomes between patients who underwent ASCT and those who only received observation after remission of induction treatment. We found that ASCT was significantly associated with favorable survival outcomes. The median overall survival (OS) of patients who underwent ASCT was not reached, with a 5-year OS rate of 80.5%, a median progression-free survival (PFS) of 77 months, and a 3-year PFS rate of 59.8%, all of which were much higher than the rates observed in patients who did not undergo transplantation. These results are consistent with previous studies (17, 18), suggesting that ASCT can serve as effective consolidation therapy following induction chemotherapy remission in patients with PTCL, leading to a significant improvement in long-term survival.

In addition to ASCT, the multivariate analysis revealed that the presence of HLH in combination was also identified as an independent prognostic factor associated with worse progression-free survival (PFS) and overall survival (OS). HLH, characterized by an ineffective immune response resulting from abnormal overactivity accompanied by an inflammatory cytokine storm, leads to multisystem damage and rapid deterioration. In patients with lymphoma, the presence of HLH indicates a poor prognosis and early mortality (19). The 30-day survival rate in the acute phase of malignancy-associated HLH ranges from 18% to 70%, and the median survival time is reported to be between 36 and 230 days; T-cell lymphoma-associated HLH carries a worse prognosis compared to B-cell lymphoma-associated HLH (20).

Additionally, to identify the population that may benefit more from ASCT, we conducted a subgroup analysis. Although limited by a small sample size, patients with stage III or IV disease, an IPI score ≥ 3, or high LDH levels may achieve better progression-free survival (PFS) and overall survival (OS) from ASCT. Similar results were reported in a previous study (18).

Pegylated liposomal doxorubicin (PLD), a novel chemotherapeutic agent with targeted anticancer properties, acts directly at the tumor site, mitigating the drawbacks associated with the high cardiotoxicity of free anthracycline drugs, thereby achieving improved safety in clinical practice. Studies have indicated that combination therapy with PLD following ASCT is effective, and its use as a bridge to transplantation is deemed safe (21). PLD has demonstrated efficacy in certain lymphomas with acceptable adverse effects, particularly benefiting elderly patients (22, 23). In our study, patients treated with PLD-containing induction chemotherapy regimens exhibited a higher overall response rate (ORR) better progression-free survival (PFS), and overall survival (OS) compared to those treated with traditional doxorubicin. This finding is consistent with the results reported by Xia (24). Studies have indicated that PLD is less extruded by pegylation and inhibits pump activity compared to Adriamycin, suggesting that PLD may be more effective against drug-resistant lymphomas than conventional Adriamycin (25). Some studies have substituted pegylated liposomal doxorubicin (PLD) for doxorubicin in CHOP regimens for the treatment of lymphoma in older patients, reporting reduced cardiotoxicity and potentially improved disease control (26, 27). Thus, PLD could be considered an effective alternative for bridging high-risk patients to transplantation.

The retrospective design of the study implies that unintentional bias and confounding variables may unavoidably exist. There are three major limitations to consider. Firstly, the patients included in this study were exclusively from our hospital, potentially introducing selection bias. Secondly, the relatively small sample size limited the statistical power of the study, particularly in subgroup analyses. Finally, the decision not to undergo ASCT was partly influenced by patients’ general condition, which could be associated with worse survival outcomes.

Overall, autologous stem cell transplantation (ASCT) can serve as a consolidation therapy following chemotherapy in PTCL patients, substantially extending long-term survival with an acceptable safety profile. Certain subgroups, particularly those with high risks, may derive greater benefits. Additionally, chemotherapy regimens containing pegylated liposomal doxorubicin (PLD) for PTCL patients may yield higher remission rates compared to conventional doxorubicin-containing chemotherapy regimens. However, due to the limitations of this study, a large multicenter randomized trial is warranted to validate these conclusions.

## Data availability statement

The raw data supporting the conclusions of this article will be made available by the authors, without undue reservation.

## Ethics statement

The studies involving humans were approved by Ethics Committee of Huadong Hospital. The studies were conducted in accordance with the local legislation and institutional requirements. The ethics committee/institutional review board waived the requirement of written informed consent for participation from the participants or the participants’ legal guardians/next of kin because Retrospective design waived individual informed consent.

## Author contributions

WF: Data curation, Formal analysis, Funding acquisition, Investigation, Methodology, Software, Visualization, Writing – original draft, Writing – review & editing. JM: Conceptualization, Investigation, Project administration, Resources, Supervision, Data curation, Writing – review & editing. MW: Funding acquisition, Investigation, Methodology, Software, Data curation, Writing – review & editing. WQ: Data curation, Methodology, Software, Writing – review & editing. PC: Data curation, Software, Validation, Writing – review & editing. YH: Data curation, Investigation, Software, Supervision, Writing – review & editing. MC: Data curation, Software, Supervision, Writing – review & editing. YuX: Data curation, Software, Writing – review & editing. ZH: Data curation, Formal analysis, Writing – review & editing. HZ: Data curation, Validation, Writing – review & editing. YaX: Conceptualization, Formal analysis, Funding acquisition, Investigation, Methodology, Project administration, Resources, Supervision, Validation, Visualization, Writing – review & editing. LS: Conceptualization, Formal analysis, Funding acquisition, Investigation, Methodology, Project administration, Resources, Supervision, Validation, Visualization, Writing – review & editing.

## References

[1] Ko YH Kim CW Park CS Jang HK Lee SS Kim SH. REAL classification of Malignant lymphomas in the Republic of korea. Cancer. (1998) 83:806. 9708949

[2] Anderson JR Armitage JO Weisenburger DD. Epidemiology of the non-Hodgkin's lymphomas: Distributions of the major subtypes differ by geographic locations. Ann Oncol. (1998) 9:717–20. doi: 10.1023/A:1008265532487 9739436

[3] D'Amore F Relander T Lauritzen GF Jantunen E Hagberg H Anderson H. Dose-dense induction followed by autologous stem cell transplant (ASCT) leads to sustained remissions in a large fraction of patients with previously untreated peripheral t-cell lymphomas (PTCLS) - overall and subtype-specific results of a phase II study f. Haematol. Hematol J. (2009) 94, 1082.

[4] Rodrıguez J Conde E Gutierrez A Reyes A Angel L Marín. Frontline autologous stem cell transplantation in high-risk peripheral T-cell lymphoma: A prospective study from The Gel-Tamo Study Group. Eur J Haematol. (2007) 79:32–8. doi: 10.1111/j.1600-0609.2007.00856.x 17598836

[5] Corradini P Tarella C Zallio F Dodero A Zanni M Valagussa P. Long-term follow-up of patients with peripheral T-cell lympho- mas treated up-front with high-dose chemotherapy followed by autologous stem cell transplantation. Leukemia. (2006) 20:1533–8. doi: 10.1038/sj.leu.2404306 16871285

[6] Mercadal S Briones J Xicoy B Pedro C Escoda L Estany C. Intensive chemotherapy (high-dose CHOP/ESHAP regimen) followed by autologous stem-cell transplantation in previously untreated patients with peripheral T-cell lymphoma. Ann Oncol. (2008) 19:958–63. doi: 10.1093/annonc/mdn022 18303032

[7] Reimer P Rudiger T Geissinger E Florian W Christoph N Norbert S. Autologous stem-cell transplantation as first-line therapy in peripheral T-cell lymphomas: Results of a prospective multicenter study. J Clin Oncol. (2009) 27:106–113. doi: 10.1200/JCO.2008.17.4870 19029417

[8] d’Amore F Relander T Lauritzsen GF Jantunen E Hagberg H Anderson H. Up-front autologous stem-cell transplantation in peripheral T-cell lymphoma: NLG-T01. J Clin Oncol. (2012) 30:3093–9. doi: 10.1200/JCO.2011.40.2719 22851556

[9] Fossard G Broussais F Coelho I Bailly S Nicolas-Virelizier E Toussaint E. Role of up-front autologous stem-cell transplantation in peripheral T-cell lymphoma for patients in response after induction: an analysis of patients from LYSA centers. Ann Oncol. (2018) 29:715–23. doi: 10.1093/annonc/mdx787 29253087

[10] Delsol G. The 2008 WHO lymphoma classification. Annales Pathol. (2008) 28 Spec No 1:S20–4. doi: 10.1016/j.annpat.2008.09.002 18984289

[11] Philip T Guglielmi C Hagenbeek A Somers R van der Lelie H Bron D. Autologous bone marrow transplantation as compared with salvage chemotherapy in relapses of chemotherapy-sensitive non-Hodgkin's lymphoma. N. Engl J Med. (1995) 333:1540–5. doi: 10.1056/NEJM199512073332305 7477169

[12] Sun Y Guo L Hu X Zhu F Yang SE. Clinical analysis of high-dose chemotherapy combined with autologous hematopoietic stem cell transplantation for treatment of lymphoma. Chin J Cancer Prev Treat. (2019) 26:963–8.

[13] Haioun C Lepage E Gisselbrecht C Salles G Coiffier B Brice P. Survival benefit of high-dose therapy in poor-risk aggressive non-Hodgkin's lymphoma: final analysis of the prospective LNH87-2 protocol–a groupe d'Etude des lymphomes de l'Adulte study. J Clin Oncol. (2000) 18:3025. doi: 10.1200/JCO.2000.18.16.3025 10944137

[14] Gui L Yuan K Xiao-hui H Ying-heng L Hong-zhi Z Xiao-hong H. High-dose therapy and autologous stem cell transplantation in peripheral T-cell lymphoma: treatment outcome and prognostic factor analysis. Int J Hematol. (2013) 99(1):69–78. doi: 10.1007/s12185-013-1465-y 24258711

[15] Shunrong S Wulipan F Lin S Min W Zilan H Wensi Q. Maintenance regimen of GM-CSF with rituximab and lenalidomide improves survival in high-risk B-cell lymphoma by modulating natural killer cells. Cancer Med. (2023) 12:12975–85. doi: 10.1002/cam4.5969 PMC10315792 37081754

[16] Casadei B Pellegrini C Tonialini L Argnani L Zinzani PL. Interesting activity of pegylated liposomal doxorubicin in primary refractory and multirelapsed Hodgkin lymphoma patients: bridge to transplant. Hematol. Oncol. (2018) 36(2):489–91. doi: 10.1002/hon.2492 29363146

[17] García-Sancho AM Bellei M López-Parra M Gritti G Cortés M Novelli S. Autologous stem-cell transplantation as consolidation of first-line chemotherapy in patients with peripheral T-cell lymphoma: a multicenter GELTAMO/FIL study. Haematologica. (2022) 107:2675–84. doi: 10.3324/haematol.2021.279426 PMC9614542 35320921

[18] Gao H Wu M Hu S Ning D Xinqiang J Lan M. Effect of autologous hematopoietic stem cell transplantation for patients with peripheral T-cell lymphoma in China: A propensity score-matched analysis. Front Oncol. (2022) 12:1039888. doi: 10.3389/fonc.2022.1039888 36465366 PMC9712948

[19] Han AR Lee HR Park BB Hwang IG Park S Lee SC. Lymphoma-associated hemophagocytic syndrome: clinical features and treatment outcome. Ann Hematol. (2007) 86:493–8. doi: 10.1007/s00277-007-0278-6 17347847

[20] Arca M Fardet L Galicier L Rivière S Marzac C Aumont C. Prognostic factors of early death in a cohort of 162 adult haemophagocytic syndrome: impact of triggering disease and early treatment with etoposide. Br J Haematol. (2015) 168:63–8. doi: 10.1111/bjh.13102 25157895

[21] Pulini S Rupoli S Goteri G Pimpinelli N Alterini R Tassetti A. Pegylated liposomal doxorubicin in the treatment of primary cutaneous T-cell lymphomas. Haematologica. (2007) 92:686–9. doi: 10.3324/haematol.10879 17488695

[22] Perea G Caballero MD Mateos MV Ribera JM de Oteyza JP Arranz R. Cyclophosphamide, pegylated liposomal doxorubicin (Caelyx), vincristine and prednisone (CCOP) in elderly patients with diffuse large B-cell lymphoma: results from a prospective phase II study. Haematologica. (2002) 87:822–7. doi: 10.3324/%x 12161358

[23] Tsavaris N ,Kosmas C Vadiaka M Giannouli S Siakantaris MP Vassilakopoulos T. Pegylated liposomal doxorubicin in the CHOP regimen for older patients with aggressive (stages III/IV) non-Hodgkin’s lymphoma. Anticancer Res. (2002) 22:1845–8. 12168880

[24] Xia Z Lv F Xue K Zhang Q Ji D Cao J. Pegylated lipsomal doxorubicin combined with cyclophosphamide, vincristine/vindesine, and prednisone in patients with aggressive T-cell lymphoma: preliminary results of a phase II study. Hematol. Oncol. (2017) 35:394–4. doi: 10.1002/hon.2439_162

[25] Abouyabis AN Shenoy PJ Sinha R Flowers CR Lechowicz MJ. A systematic review and meta-analysis of front-line anthracycline-based chemotherapy regimens for peripheral T-cell lymphoma. Int Scholarly Res Notices. (2011) 2011:1–14. doi: 10.5402/2011/623924 PMC3197255 22084700

[26] Riganti C Voena C Kopecka J Corsetto PA Montorfano G Enrico E. Liposome-encapsulated doxorubicin reverses drug resistance by inhibiting P-glycoprotein in human cancer cells. Mol Pharmaceut. (2011) 8:683–700. doi: 10.1021/mp2001389 21491921

[27] Visani G Guiducci B D"Adamo F Mele A Nicolini G Leopardi G. Cyclophosphamide, pegylated liposomal doxorubicin, vincristine and prednisone (CDOP) plus rituximab is effective and well tolerated in poor performance status elderly patients with non-Hodgkin lymphoma. Leukemia Lymphoma. (2005) 46:477–9. doi: 10.1080/10428190400013688 15621843

